# Investigating tumor-infiltrating lymphocytes as predictors of lymph node metastasis in deep submucosal invasive esophageal squamous cell carcinoma: a retrospective cross-sectional study

**DOI:** 10.1007/s00535-025-02286-0

**Published:** 2025-07-25

**Authors:** Hirona Konishi, Yuji Urabe, Yoshiki Hatsushika, Satoshi Masuda, Takeo Nakamura, Kazuki Ishibashi, Junichi Mizuno, Takeshi Takasago, Hidenori Tanaka, Akiyoshi Tsuboi, Ken Yamashita, Yuichi Hiyama, Yoshihiro Kishida, Hidehiko Takigawa, Akira Ishikawa, Toshio Kuwai, Yoichi Hamai, Yuji Murakami, Shiro Oka

**Affiliations:** 1 Department of Gastroenterology, Hiroshima University Hospital, 1-2-3, Kasumi, Minamiku, Hiroshima, 734-8551 Japan; 2 Department of Clinical Research Center, Hiroshima University Hospital, Hiroshima, Japan; 3 Department of Molecular Pathology, Graduate School of Biomedical and Health Sciences, Hiroshima University, Hiroshima, Japan; 4 Department of Gastrointestinal Endoscopy and Medicine, Hiroshima University, Hiroshima, Japan; 5 Department of Surgical Oncology, Hiroshima University, Hiroshima, Japan; 6 Department of Radiation Oncology, Graduate School of Biomedical and Health Sciences, Hiroshima University, Hiroshima, Japan

**Keywords:** Esophageal cancer, Lymph node metastasis, Tumor-infiltrating T-cells, Tumor microenvironment

## Abstract

**Background:**

Various subtypes of tumor-infiltrating lymphocytes (TILs) are associated with prognosis in various cancer types. In esophageal squamous cell carcinoma (ESCC), TILs have been associated with prognosis in advanced stages of the disease. However, their significance in superficial esophageal squamous cell carcinoma (SESCC) remains unknown. In this study, we investigated the role of TILs in SESCC.

**Methods:**

First, we included 212 SESCC lesions with a diameter of 10–20 mm (65 pT1a-EP, 74 pT1a-LPM,40 pT1a-MM, and 33 pT1b-SM lesions) that were resected at our hospital from November 2007 to December 2019. We then examined changes in TILs related to tumor invasion. We evaluated the phenotype and number of TILs using triple immunofluorescence staining for CD4, CD8, and FoxP3. In addition, we selected 97 consecutive pT1b-SM lesions treated during the same period. These specimens were used to examine the association between TILs and lymph node metastasis (LNM) in pT1b-SM cases.

**Results:**

The number of CD4 + , CD8 + , and FoxP3 + TILs infiltrating the tumor increased significantly with increasing invasion depth. In pT1b-SM lesions, CD4 + , CD8 + , and FoxP3 + TIL counts were significantly higher in the invasive front (IF) than in the tumor center (CT). Moreover, in patients undergoing surgical resection or endoscopic submucosal dissection (regardless of additional chemoradiotherapy), the number and ratio of FoxP3 + TILs in IF were significantly higher in patients with LNM than in those without, suggesting their potential utility as predictive biomarkers.

**Conclusions:**

The number and ratio of FoxP3 + TILs in the IF may be an indicator of LNM risk in SESCC.

**Supplementary Information:**

The online version contains supplementary material available at 10.1007/s00535-025-02286-0.

## Introduction

Esophageal squamous cell carcinoma (ESCC) is one of the most common malignant tumors in East Asia [1]. Notably, esophageal cancer is also among the most aggressive malignancies. Even at an early stage, it typically exhibits lymph node metastasis (LNM) and invasion into adjacent organs. In particular, the 5-year survival rate of esophageal cancer is only 16.9%, even with current advanced medical treatment [2].

Superficial ESCC (SESCC) is defined as a tumor confined to the epithelium (EP) and extending up to the submucosa (SM) but not invading the muscularis propria (MP). According to the Japan Esophageal Society (JES) 2022 guideline [3], endoscopic resection is recommended as the standard treatment for intra-mucosal carcinoma. However, surgical resection with lymphadenectomy or chemoradiotherapy (CRT) is recommended for T1b-SM, which has a high LNM rate. Clinical factors known to affect the risk of LNM in esophageal cancer include tumor depth, differentiation, size, and the presence of vascular invasion. However, LNM does not occur in all cases exhibiting these factors. Accurate identification of cases with a high risk of LNM could contribute to reducing medical costs and minimizing unnecessary invasive treatments for patients [4, 5]. Therefore, stratifying the risk of LNM is important for ensuring appropriate treatment choices.

In the tumor microenvironment, monocytes, dendritic cells, macrophages, and lymphocytes are intricately involved in the antitumor response [6, 7]. Various tumor-infiltrating lymphocyte (TIL) subtypes have been identified, each of which plays an important role and is associated with prognosis in various types of cancer. In lung cancer, the presence of high levels of CD4 + and CD8 + T-cells is an important indicator of improved prognosis [8–10]. In colorectal cancer and ESCC, CD8 + and FoxP3 + T-cells are reportedly associated with prognosis [11–14]. Although these previous studies have primarily focused on advanced cancer, a recent study reported that FoxP3 + counts and their ratios are associated with tumor invasion and LNM in early-stage colorectal cancer [15].

Generally, tumor depth and lymphovascular invasion are considered risk factors for LNM in T1b ESCC. However, no studies exploring the relationship between tumor microenvironment and LNM are available. Furthermore, no study has examined the changes in the tumor microenvironment in SESCC with different degrees of invasion, that is, epithelial (EP), lamina propria mucosal (LPM), muscularis mucosal (MM), and submucosal (SM) cancers. Therefore, the present study aimed to analyze the number and ratio of TILs in relation to the risk of LNM and evaluate their clinical significance as a determinant of additional treatment in SESCC.

## Methods

### Study population

First, we included 212 consecutive SESCC cases with a diameter of 10–20 mm (65 pT1a-EP, 74 pT1a-LPM, 40 pT1a-MM, and 33 pT1b-SM lesions) that were endoscopically or surgically resected at our hospital from November 2007 to December 2019. We then examined the changes in TILs related to tumor invasion. We evaluated the phenotype and number of TILs using triple immunofluorescence staining for CD4, CD8, and FoxP3.

Next, we selected 97 consecutive pT1b-SM lesions treated surgically or endoscopically during the same period. In this study, surgical treatment cases were defined as those in which patients underwent surgery alone as well as those in which patients received additional surgical intervention following endoscopic submucosal dissection (ESD). In contrast, cases that did not receive any additional treatment or underwent additional CRT following endoscopic therapy were categorized as endoscopic treatment cases. As a general policy of our hospital, additional treatment for pT1b-SM cases was recommended in the form of either CRT or surgery. However, the decision to forego additional treatment was occasionally made based on the patient's general condition and preferences. The choice between CRT and surgery was determined according to the patient's wishes.

The association between TILs and LNM was investigated using the pT1b-SM specimens from each of the surgical and endoscopic treatment groups. All clinical data were obtained by retrospectively reviewing patient records. LNM was defined as pathological recurrence detected during the 5-year follow-up in endoscopically treated cases and surgically resected cases or as pN1 in surgically resected cases. The 33 patients with pT1b-SM analyzed in the first half overlapped with the 97 patients analyzed in the second half.

### Histological evaluation

All specimens were evaluated by a pathologist according to the latest World Health Organization classification criteria [16]. The depth of the submucosa was determined following the General Rules for Clinical and Pathological Studies on Cancer of the Esophagus as outlined by JES 2022 guidelines [3]. Lesions were classified as T1a (EP/LPM/MM) or T1b (SM) carcinomas. The subclassification of pT1b-SM was based on the JSECR 2022 guidelines. In surgical specimens, the submucosa was divided into three equal layers: tumors confined to the upper one-third were defined as T1b-SM1, those in the middle one-third as T1b-SM2, and those extending to the lower one-third as T1b-SM3. In contrast, in ESD specimens, tumors with submucosal invasion measuring ≤ 200 μm from the muscularis mucosae were defined as T1b-SM1, whereas those with invasion exceeding 200 μm were classified as T1b-SM2. Lymphovascular invasion was assessed using only hematoxylin and eosin staining until October 2013 and subsequently using Elastica van Gieson and D2-40, in addition to hematoxylin and eosin. The investigator analyzing the triple immunofluorescence quantitative data was blinded to the clinical data. Subsequently, another investigator evaluated the quantitative data following integration with the clinical data.

### Assessment of phenotype, number, and distribution of TILs

**Fig. 1:**
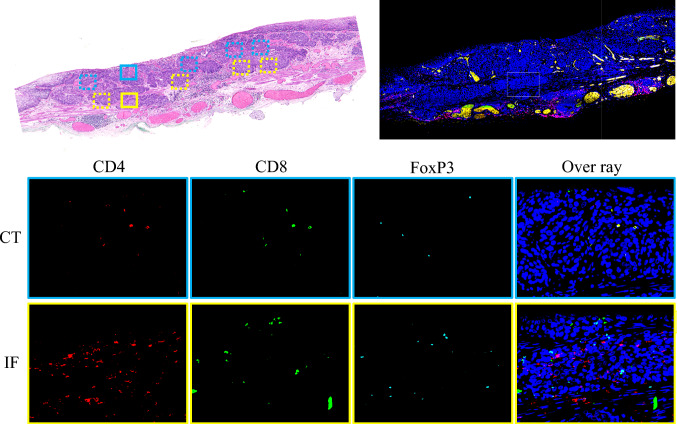
Phenotype and distribution of TILs. The number of TILs was counted in two different regions: the IF (yellow areas) and CT (blue areas). Triple immunofluorescence for CD4 (red), CD8 (green), and Foxp3 (light blue) cells. Nuclei were stained with DAPI (blue), and the overlay depicts fluorescence detected by all channels. Scale bars, 50 μm. TILs, tumor-infiltrating lymphocytes; IF, invasive front of the tumor; CT, center of the tumor; DAPI, diamidino-2-phenylindole

The phenotype, number, and distribution of TILs were evaluated using triple immunofluorescence staining for CD4, CD8, and FoxP3. The distribution of TILs was assessed by dividing the tumor tissue into two parts: the invasive front of the tumor (IF) and the center of the tumor (CT) (Fig. 1). Specimens were observed under a fluorescence microscope (BZ-X810; Keyence, Osaka, Japan). Five high-power fields (HPF; at 400 × magnification) showing substantial infiltration of TILs were selected and photographed for each sample. The numbers of CD4 +, CD8 +, and FoxP3 + T-cells were counted in each captured image using an image analyzer (BZ-H4A; Keyence). The mean number of CD4, CD8, and FoxP3 + T-cells was compared among groups relative to LNM.

### Immunofluorescence staining

Triple immunofluorescence was performed using the Opal 4-color manual immunohistochemistry (IHC) kit (NEL810001KT; PerkinElmer, Waltham, MA, USA). All specimens were fixed in 10% formaldehyde and embedded in paraffin according to routine procedures at the Department of Clinical Pathology, Hiroshima Universal Hospital (Hiroshima, Japan). A 4-μm section was cut from each specimen and deparaffinized, followed by heat-induced antigen retrieval for 15 min using microwave after-tissue pretreatment with 0.3% H_2_O_2_. The sections were then probed with an anti-CD4 antibody (1:400; ab133616; Abcam, Cambridge, UK) overnight at 4 °C. Subsequently, the sections were washed and incubated with the respective secondary antibodies for 15 min at 25 °C (room temperature), followed by washing and incubation at 25 °C for 10 min with the Opal 570 reagent. After another round of heat-induced antigen retrieval, the sections were incubated with an anti-CD8 alpha antibody (1:500; ab93278; Abcam) overnight at 4 °C. The sections were then washed and incubated with secondary antibodies for 15 min at 25 °C, followed by washing and incubation with the Opal 520 reagent at 25 °C for 10 min. After another round of heat-induced antigen retrieval, the sections were incubated with a recombinant anti-FoxP3 antibody (1:500; ab20034; Abcam) overnight at 4 °C. They were then washed and incubated with secondary antibodies for 15 min at 25 °C, followed by washing and incubation with the Opal 690 reagent at 25 °C for 10 min. After washing, the sections were counterstained with DAPI (1:500) for 5 min and were then mounted in mounting medium (Fluoromount; Diagnostic BioSystems, Pleasanton, CA, USA).

### Ethics statement

The study was conducted in accordance with the principles of the Declaration of Helsinki and approved by the Institutional Review Board of Hiroshima University (E2024-0096). Although the Ethics Committee of Hiroshima University Hospital waived the requirement for informed consent owing to the use of anonymized data, informed consent was obtained from the patients using a consent form; the patients could withdraw from the study using an opt-out option.

### Statistical analyses

Statistical analyses were performed using the JMP version 16.0 software (SAS Institute Inc., Cary, NC, USA). Quantitative data are presented as the mean ± standard deviation or as percentages and were compared using Pearson’s chi-square or Fisher’s exact test. Continuous variables were analyzed using the non-parametric method. Statistical significance was set at *P* < 0.05. The area under the curve (AUC) of the receiver operating characteristics (ROC) was calculated using JMP. The ROC curve was generated by plotting the true positive rate (sensitivity) against the false positive rate (1-specificity) at various threshold settings. The AUC was computed to evaluate the classification performance of the model, with values close to 1 indicating good discrimination.

## Results

### Changes in the number, phenotype, and distribution of TILs during ESCC progression

The clinicopathological features of SESCC lesions stratified by invasion depth (pT1a-EP, pT1a-LPM, pT1a-MM, and pT1b-SM) are presented in Online Resource 1.

Significant differences were observed among the groups in tumor size, macroscopic type, tumor location, treatment method, and the rates of venous and lymphatic invasion. In contrast, no significant differences were observed in age, sex, tumor circumferential involvement, or the rate of clinical LNM among the groups.

**Fig. 2:**
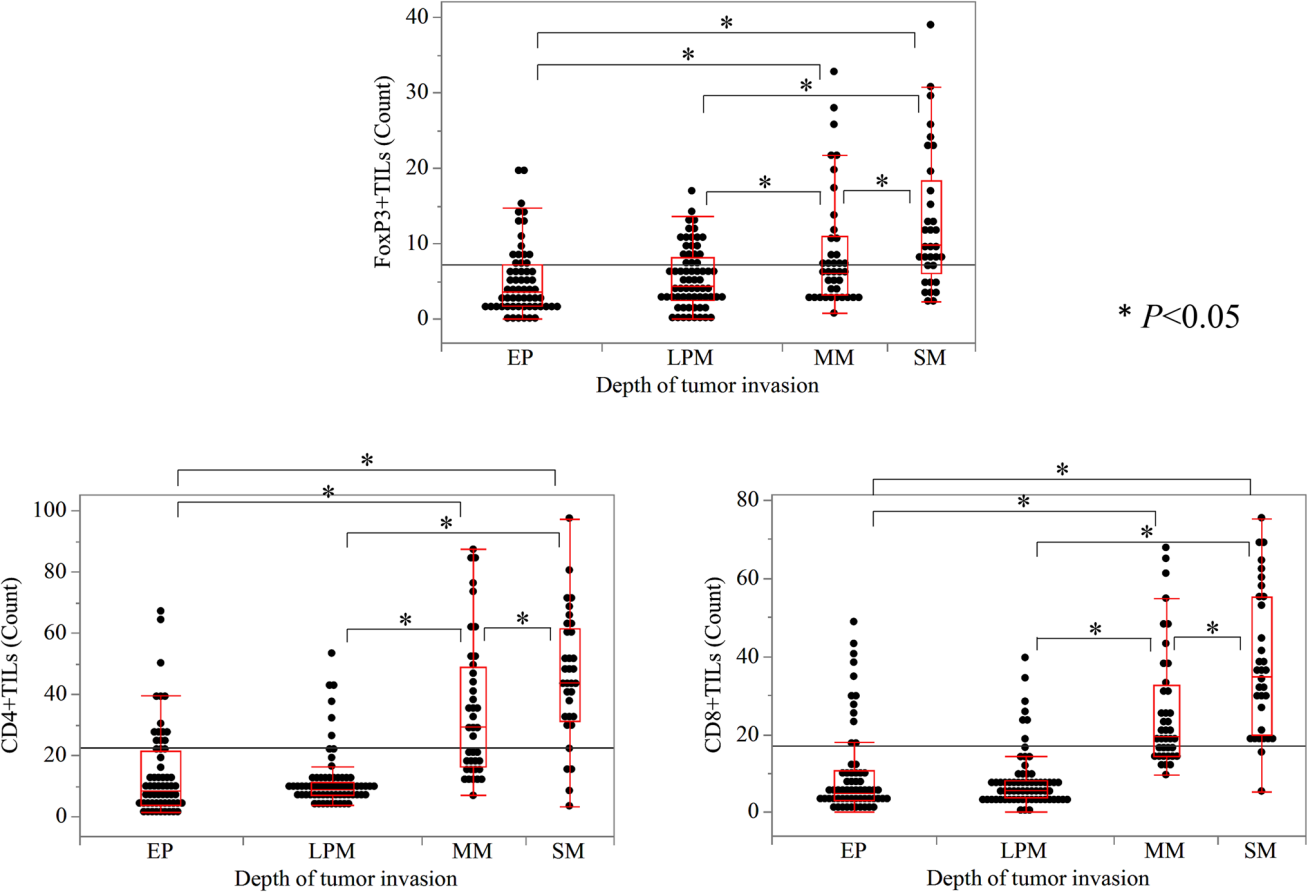
Comparison of the counts of TILs according to the progression of SESCCs in the IF in the pT1a-EP, pT1a-LPM, pT1a-MM, and pT1b-SM groups. The number of HPF significantly increased as the depth increased (*P* < 0.05). TILs, tumor-infiltrating lymphocytes; IF, invasive front of the tumor; EP, epithelium; MM, muscularis mucosae; SM, submucosa

In addition, TILs were evaluated in each group (Fig. 2). The number of TILs per HPF significantly increased with increasing depth of invasion, except between pT1a-EP and pT1a-LPM lesions (*P* < 0.05).

### Clinicopathological characteristics of patients with PT1b-SM cases

The clinicopathological features of T1b-SM cases according to the depth of submucosal invasion (pT1b-SM1 vs. pT1b-SM2/3) are summarized in Online Resource 2. Surgical treatment alone was more frequently performed in the pT1b-SM2/3 group than in the pT1b-SM1 group. In addition, LNM and venous invasion were significantly more frequent in the pT1b-SM2/3 group than in the pT1b-SM1 group (30% vs. 7%, *P* = 0.01; 33% vs. 7%, *P* < 0.01, respectively). However, there was no significant difference in the rate of lymphatic invasion between the two groups (*P* = 0.71).

A total of 38 (39%) patients underwent surgical resection, whereas 59 (61%) received ESD. Among these, 38 patients received additional CRT treatment, and 7 patients underwent additional surgical intervention. No LNM recurrence was observed in surgical cases with pN0.

### Association between the distribution of TILs and LNM in pT1b-SM cases

**Fig. 3:**
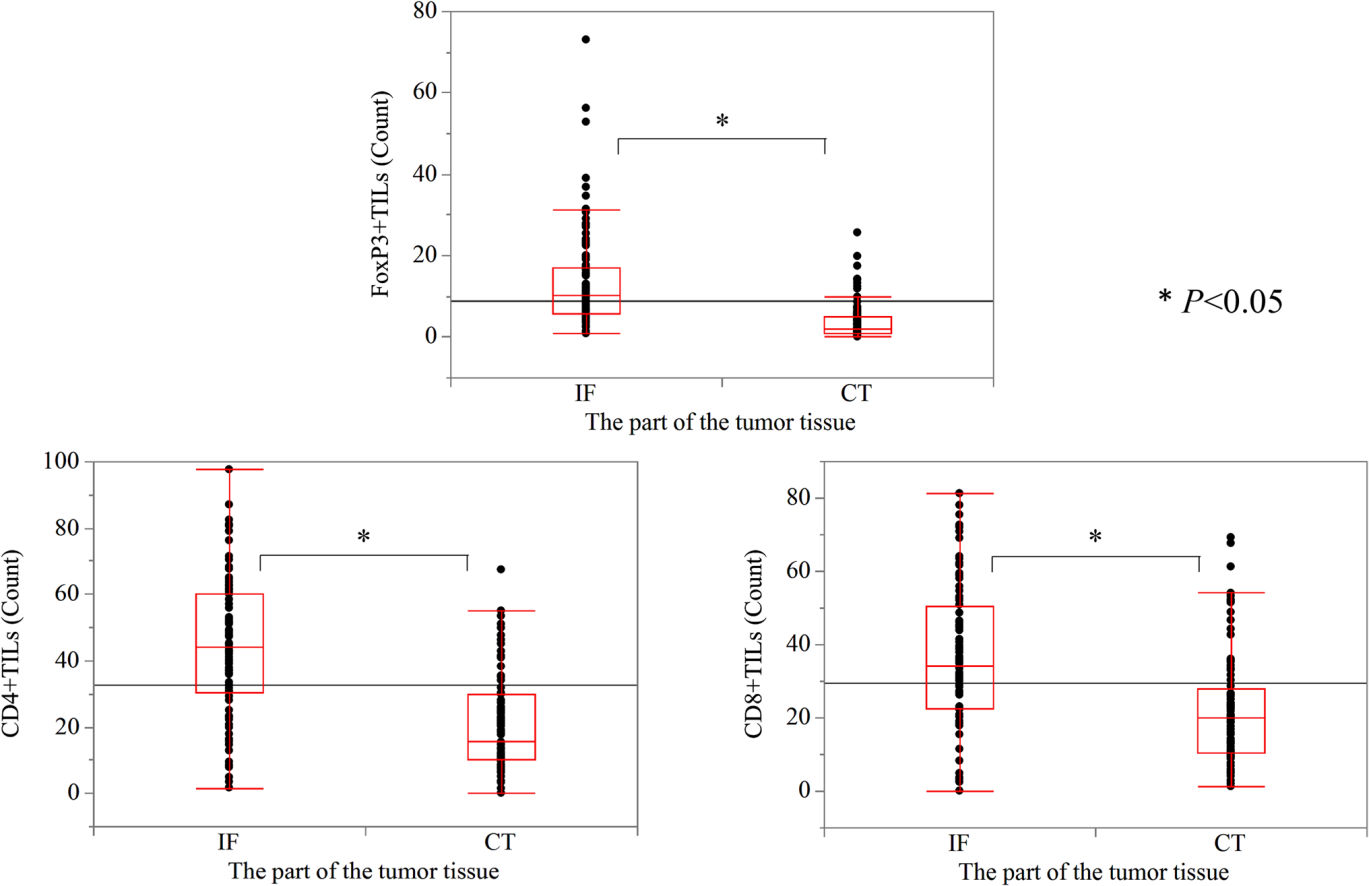
Comparison of the counts of TILs according to the progression of T1b SESCCs. The numbers of TILs in the CT (the right side of the bar graph) were compared to those in the IF (the left side of the bar graph). T1b SESCCs had significantly higher numbers of CD4, CD8, and Fopx3 cells in the IF than in the CT. TILs, tumor-infiltrating lymphocytes; SESCC, superficial esophageal squamous cell carcinoma; CT, center of the tumor; IF, invasive front of the tumor

**Fig. 4:**
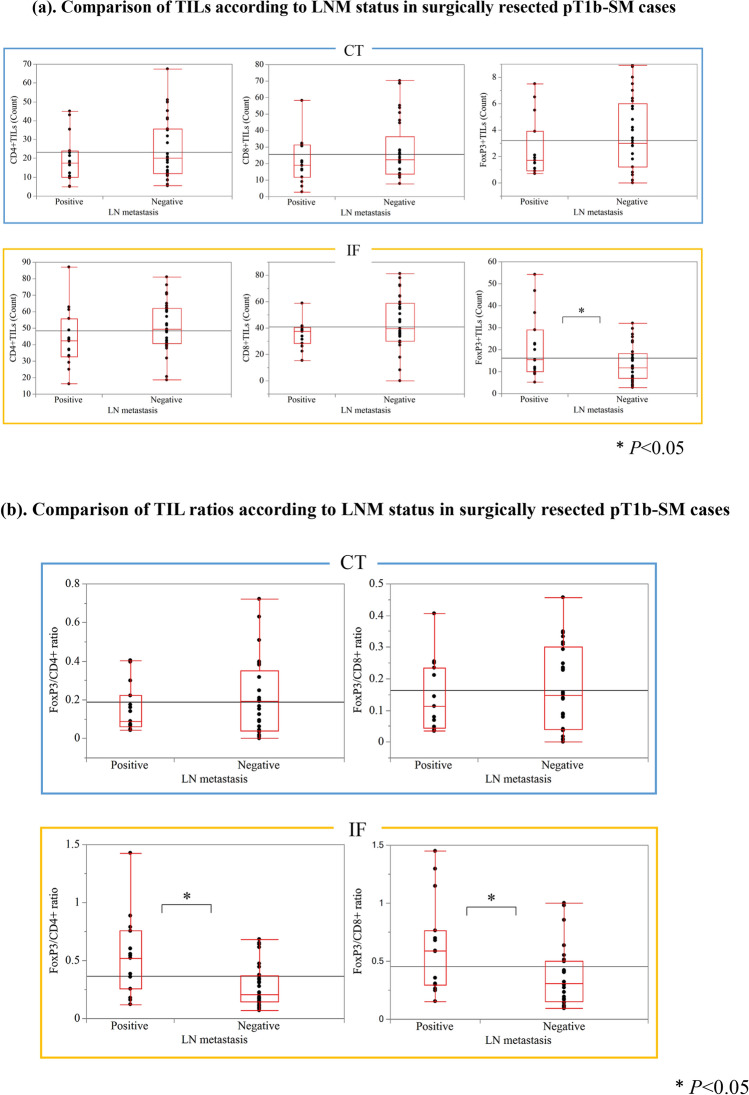
Comparison of TILs according to LNM status in surgically resected pT1b-SM cases. **a** No significant difference was observed in terms of CD4 +, CD8, and FoxP3 + T cell counts in the CT between patients with and without LNM. In the IF, there was no significant difference in CD4 + and CD8 + T cell count, although FoxP3 + T-cells were significantly more numerous in the LN-positive cases. **b** No significant difference was observed in the ratio of FoxP3/CD4 to FoxP3/CD8 in the CT between patients with and without LNM. Patients with LNM had significantly higher FoxP3/CD4 and FoxP3/CD8 ratios at the IF than patients without LNM. TILs, tumor-infiltrating lymphocytes; SESCC, superficial esophageal squamous cell carcinoma; LNM, lymph node metastasis; CT, center of the tumor; IF, invasive front of the tumor

We also evaluated the number of CD4 +, CD8 +, or FoxP3 + T-cells in the CT and IF regions in pT1b-SM cases (Fig. 3). The numbers of CD4 +, CD8 +, and Fopx3 + T-cells were all significantly higher in the IF than in the CT (*P* < 0.01). Subsequently, we compared the number of TILs between patients with and without LN metastases (Fig. 4). In the CT, no significant differences were observed in the numbers of CD4 +, CD8 +, or FoxP3 + TILs. Similarly, in the IF, no significant differences were observed in the numbers of CD4 + and CD8 + TILs; however, the number of FoxP3 + T-cells was significantly higher in the group with LNM than in that without LNM (*P* < 0.05). Furthermore, in the CT, no significant differences in the FoxP3/CD4 and FoxP3/CD8 ratios were detected. In contrast, in the IF, the FoxP3/CD4 and FoxP3/CD8 ratios were significantly high in LNM cases (*P* < 0.05).

### Association between TILs and LNM in surgical cases

**Fig. 5:**
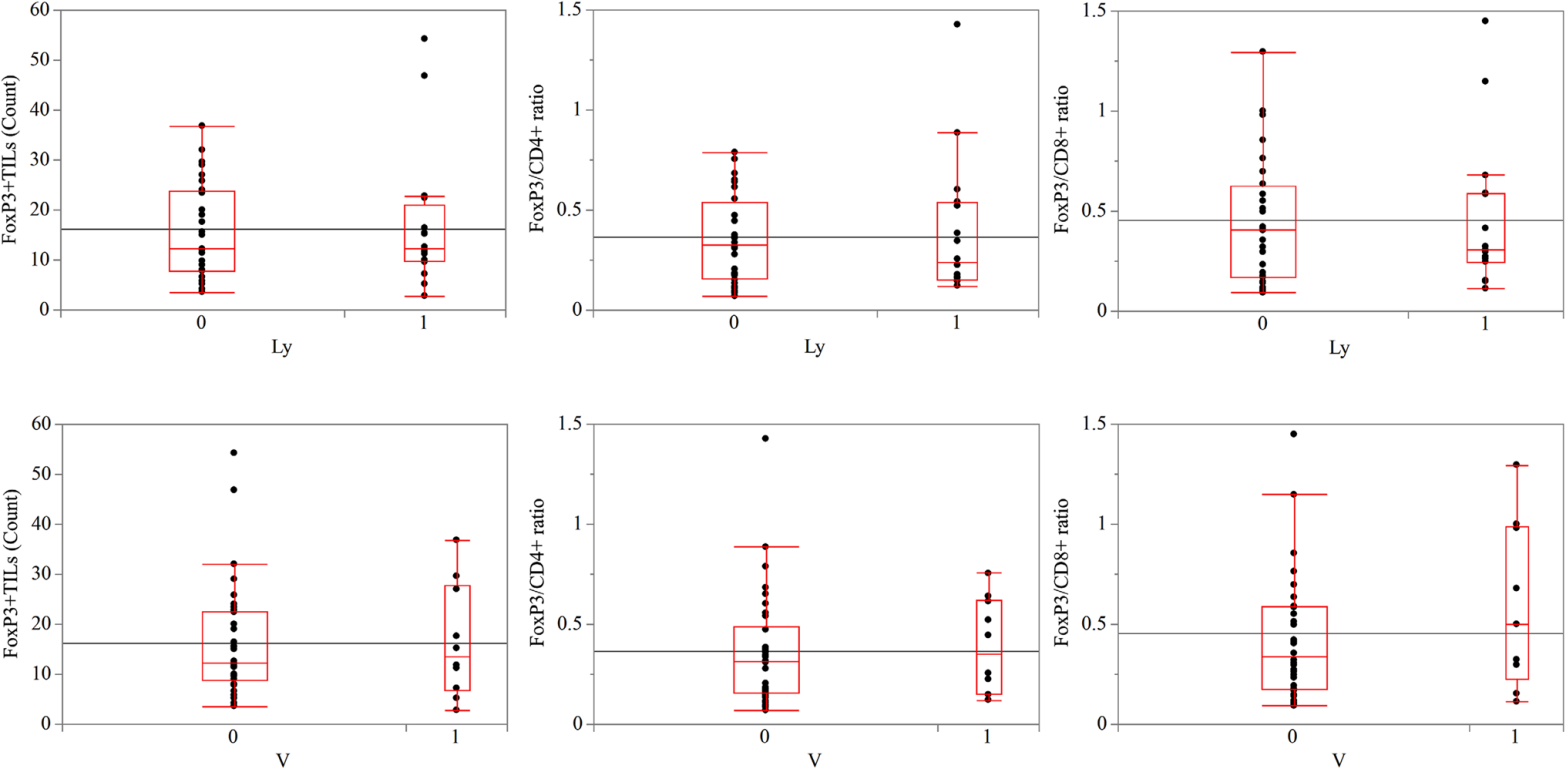
Comparison of TIL counts in the IF according to lymphovascular invasion in surgically resected T1b SESCC cases. No significant difference was observed in terms of FoxP3 + and the ratio of FoxP3/CD4 + and FoxP3/CD8 + T cell counts according to lymphovascular invasion. TILs, tumor-infiltrating lymphocytes; SESCC, superficial esophageal squamous cell carcinoma; IF: invasive front; LNM, lymph node metastasis

For 45 out of the 97 patients with pT1b-SM lesions who underwent surgery, we investigated the association of TILs with LNM; the clinicopathologic characteristics of patients with T1b-SM are presented in Online Resource 3. Of the 45 patients with pT1b-SM who underwent surgery, only two were clinically diagnosed as N1, whereas the others were diagnosed as N0. In total, 15 patients were included in the group with LNM, whereas 30 were classified as without LNM. In terms of lesion depth, among patients with LNM, 7% had SM1, whereas 93% had SM2/SM3. Among patients without LNM, 30% had SM1, whereas 70% had SM2/SM3; no significant differences were observed between the two groups. Regarding lymphovascular invasion, among patients with LNM, 27% had positive venous invasion, whereas 67% had positive lymphatic invasion; among those without LNM, 20% had positive venous invasion, whereas 20% had positive lymphatic invasion. This finding indicates that the group with LNM had significantly more lesions with positive lymphatic invasion than the group without LNM. We then compared the counts of TILs according to lymphovascular invasion in patients with T1b SESCC who underwent surgery (Fig. 5). We did not detect any differences in the number of FoxP3 + TILs or in the FoxP3/CD4 and FoxP3/CD8 ratios in IF between the two groups.

The AUC of the FoxP3 + TILs in the IF was 0.65, with an optimal cutoff value of 8, sensitivity of 34%, and specificity of 28%. The AUC of the FoxP3/CD4 + TIL ratio in the IF was 0.74, with an optimal cutoff value of 0.34, sensitivity of 72%, and specificity of 46%. The AUC of the FoxP3/CD8 + TIL ratio in the IF was 0.73, with an optimal cutoff value of 0.55, sensitivity of 86%, and specificity of 46%.

### Association between TILs and LNM in ESD cases

We also examined the association between TILs and LNM in 52 endoscopic treatment cases, comprising patients who underwent observation without additional therapy after ESD and those who received additional CRT after ESD. The clinicopathological features of these patients are presented in Online Resource 4. All these cases were clinically diagnosed as N0. During follow-up, LNM was observed in 7 cases. Comparison of the clinicopathological features between patients with and without LNM revealed that the presence of lymphatic invasion was significantly more frequent in the LNM group than in the group without LNM (*P* = 0.04). However, no other significant differences were identified between the two groups.

**Fig. 6:**
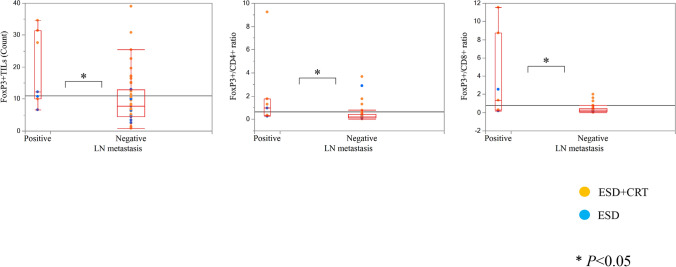
Comparison of the counts and ratios of TILs in IF according to the status of lymph node metastasis in T1b SESCC specimens resected via endoscopic submucosal dissection. Patients with LNM had significantly higher FoxP3 + T-cells and FoxP3/CD4 and FoxP3/CD8 ratios in the IF than patients without LNM. TILs, tumor-infiltrating lymphocytes; SESCC, superficial esophageal squamous cell carcinoma; IF: invasive front; LNM, lymph node metastasis

We also evaluated the distribution of TILs in the IF in relation to the LNM status, even in patients with T1b-SM who underwent ESD (Fig. 6). Notably, we again observed differences in the number of FoxP3 + TILs and the FoxP3/CD4 and FoxP3/CD8 ratios within the IF between the two groups (*P* < 0.05). However, we typically observed outliers without LNM. Almost all outlier cases were treated with CRT after ESD.

## Discussion

In the present study, to evaluate the role of TILs in SESCC, we performed triple immunostaining for CD4, CD8, and FoxP3. Our results revealed that the number of TILs was significantly increased in the region of deeper tumor invasion. In addition, the increased number and ratio of FoxP3 TILs in the IF correlated with an elevated rate of LNM in pT1b-SM cases. Despite the increased number of outliers among ESD cases, most of the outliers were cases in which CRT was performed as an additional treatment following ESD.

Various studies have explored the relationship between the microenvironment and TILs in various types of cancer, including esophageal cancer. However, most of these previous studies have focused on advanced cancers, with none focusing on SESCC. In addition, although TILs have been compared between the CT and IF in early colorectal cancer [15], to the best of our knowledge, the present study is the first to describe such findings in SESCC. Specifically, in this study, we investigated the changes in TILs in SESCC. Moreover, as LNM is an important indicator of cancer progression and is closely related to the function of the immune system, we examined the distribution of TILs in pT1b-SM lesions with or without LNM to evaluate the clinical significance of TILs as a determinant of additional treatment in SESCC.

TILs play a pivotal role in tumor invasion by inducing angiogenesis and apoptosis. The presence of TILs is generally considered an indication of an active immune response against the tumor. A high number of TILs suggests an active immune response [17, 18]. In this study, we evaluated the number of CD4 +, CD8 +, and FoxP3 + TILs in pT1a-EP, pT1a-LPM, pT1a-MM, and pT1b-SM, in the CT and IF regions in pT1b-SM cases. We found that the number of TILs per HPF significantly increased with increasing depth of invasion, except between pT1a-EP and pT1a-LPM lesions. Moreover, the numbers of CD4 +, CD8 +, and Fopx3 + T-cells were significantly higher in the IF than in the CT. These findings suggest that greater tumor invasion depth and a more pronounced IF are associated with a stronger immune response elicited against cancer cells. The increase in the number of TILs in proportion to the degree of infiltration may represent a natural defense process of the immune system. A previous study also indicated that the number of CD4 +, CD8 +, and Fopx3 + T-cells is significantly increased with the increasing invasiveness of colorectal cancer in both early and advanced cancer cases [15]. To the best of our knowledge, our study is the first to report such findings in SESCC.

CD4 +, CD8 +, and Foxp3 + TILs play an important role in the immune system, particularly in cancer progression and LNM. CD4 is a molecule primarily expressed on the surface of helper T-cells, which helps activate other immune cells such as macrophages and B-cells [19–22]. In cancer immune responses, CD4 + TILs play a role in supporting the activity of CD8 + TILs in attacking cancer cells. In LNM, dysfunction or suppression of CD4 + TILs may contribute to cancer progression. CD8 is a molecule expressed primarily on the surface of cytotoxic T-cells. It plays a role in the direct recognition and destruction of virally infected and tumor cells by this specific T cell population [19, 22, 23]. In cancer, CD8 + TILs can recognize and kill tumor cells, actively suppressing LNM. However, cancer cells may suppress the activity of CD8 + TILs and promote LNM through various mechanisms. Foxp3 is a specific transcription factor of regulatory T-cells (Tregs), which play a role in suppressing immune responses [24–26]. In cancer, Tregs typically suppress tumor immune responses and contribute to cancer progression and LNM. In particular, increased numbers of Foxp3 + TILs in the tumor microenvironment are associated with suppressed immune responses and increased risk of LNM. Taken together, these findings suggest that a decrease in the number of CD4 + and CD8 + TILs leads to a reduction in tumor immune surveillance, enabling cancer cells to metastasize to LNs [19].

An increase in the number of FoxP3 + TILs is associated with a decrease in the number and function of CD8 + TILs, suggesting that an elevated proportion of FoxP3 + TILs may contribute to LNM [27, 28]. In patients with advanced cancer, cases with an elevated CD4/CD8 ratio are more frequently associated with LNM, distant metastasis, and poorer prognosis. Moreover, LNM is more commonly observed in cases with an elevated number and proportion of FoxP3 + TILs [13, 29]. The relationship between the tumor microenvironment, TILs, and prognosis has been reported across various types of cancers [9, 10, 30–34]. In this study, we found that the rate of LNM was significantly increased in cases with an elevated number of FoxP3 + TILs at the IF and those with increased FoxP3/CD4 and FoxP3/CD8 ratios. In contrast, although LNM was significantly more frequent in cases with lymphatic invasion, no significant correlation was observed between the presence of lymphatic invasion and either the number or the ratio of FoxP3 + TILs in the IF. These findings suggest that lymphatic invasion and an increase in FoxP3 + TILs may contribute to LNM through distinct mechanisms. Moreover, FoxP3 + TILs may play a more critical role in the “settlement phase” of LNM rather than in the initial step of vascular invasion [35], and elucidating these fundamental mechanisms remains an important subject for future research.

In the present study, in pT1b-SM cases treated with ESD alone or with additional CRT after ESD, some of the cases without the recurrence of LNM exhibited increased numbers of FoxP3 + TILs and elevated FoxP3/CD4 and FoxP3/CD8 ratios. Most of these outlier cases had received CRT after ESD. These findings suggest that CRT may have suppressed LNM recurrence and that the number and ratio of FoxP3 + TILs after ESD may serve as useful indicators for determining the necessity of additional treatment, such as CRT.

However, this study had some limitations. First, this was a retrospective study conducted at a single institution, and the sample size was relatively small. It is also essential to predict LNM separately in patients with T1a-MM, T1b-SM1, SM2, and SM3 tumors. However, in this study, LNM occurred in one pT1a-MM case and two pT1b-SM1 cases; therefore, we could not assess the association between LNM and TILs in pT1a-MM/pT1b-SM1 cases. Further studies are warranted to specifically examine whether the number or infiltration density of FoxP3 + TILs is associated with LNM in tumors exhibiting lymphatic invasion. Therefore, future prospective multicenter studies involving a large number of cases should be conducted. Second, this study focused on the relationship between clinical data and fluorescent immunostaining, and the detailed function of Fopx3 + TILs in cancer or normal tissues was not assessed. Therefore, future studies should explore these issues. In addition, CD4 +, CD8 +, and FoxP3 + TILs were examined using a single antibody. However, various subtypes of CD4 +, CD8 +, and FoxP3 + cells exist, warranting comprehensive studies using multiple antibodies in the future.

## Conclusion

In this study, the number and ratio of FoxP3 + TILs in the IF of the tumor were suggested to be possible indicators of LNM risk in SESCC. In recent years, owing to the increasingly aging population of Japan, efforts have been made to expand the indications for endoscopic treatment, a minimally invasive therapy, in the management of ESCC [36]. However, expanding the indications for endoscopic treatment to include pT1b-ESCC, which is associated with LNM, requires a more accurate stratification of LNM risk. Therefore, the findings of this study may guide the development and use of specified biomarkers for predicting LNM in pT1b-SM cases.

## Supplementary Information

Below is the link to the electronic supplementary material.

Supplementary file1 (DOCX 38 KB)
