# Supplementary material for: Investigating tumor-infiltrating lymphocytes as predictors of lymph node metastasis in deep submucosal invasive esophageal squamous cell carcinoma: a retrospective cross-sectional study
Source: J Gastroenterol. 2025 Jul 25;60(11):1362–71. doi: 10.1007/s00535-025-02286-0 (PMC12549430; doi:10.1007/s00535-025-02286-0)
Supplement: Supplementary file 1 — Supplementary file1 (DOCX 38 KB) [file 535_2025_2286_MOESM1_ESM.docx]

Supplementary table 1. Clinicopathological characteristics of patients and lesions with SESCCs

| **Characteristics** | **pT1a-EP**  **n=65** | | **pT1a-LPM**  **n=74** | | **pT1a-MM**  **n=40** | | **pT1b-SM**  **n=33** | | ***P*-value** |
| --- | --- | --- | --- | --- | --- | --- | --- | --- | --- |
| Age, mean±SD, years | 67±8 | | 68±10 | | 66±10 | | 66±14 | | 0.69 |
| Sex, male (%) | 56 | (86) | 61 | (82) | 33 | (83) | 29 | (88) | 0.85 |
| Tumor size, mm, mean ± SD | 17±3 | | 18±2 | | 16±5 | | 15±5 | | <0.01 |
| Macroscopic type (%) |  |  |  |  |  |  |  |  | <0.01 |
| 0-I | 0 | (0) | 0 | (0) | 0 | (0) | 2 | (6) |  |
| 0-Ⅱa | 2 | (3) | 1 | (1) | 2 | (5) | 2 | (6) |  |
| 0-Ⅱb | 8 | (12) | 1 | (1) | 0 | (0) | 0 | (0) |  |
| 0-Ⅱc | 55 | (85) | 72 | (98) | 38 | (95) | 29 | (88) |  |
| Tumor location (%) |  |  |  |  |  |  |  |  | 0.02 |
| Ce/Ut | 12 | (18) | 18 | (24) | 7 | (17) | 9 | (27) |  |
| Mt | 33 | (51) | 31 | (42) | 27 | (68) | 22 | (67) |  |
| Lt/Ae | 20 | (31) | 25 | (34) | 6 | (15) | 2 | (6) |  |
| Circumferential range (%) |  |  |  |  |  |  |  |  | 0.52 |
| <2/3 | 64 | (98) | 74 | (100) | 40 | (100) | 33 | (100) |  |
| ≥2/3 | 1 | (2) | 0 | (0) | 0 | (0) | 0 | (0) |  |
| Whole circumference | 0 | (0) | 0 | (0) | 0 | (0) | 0 | (0) |  |
| Treatment (%) |  |  |  |  |  |  |  |  | <0.01 |
| Surgery alone | 0 | (0) | 0 | (0) | 0 | (0) | 11 | (33) |  |
| Surgery after ESD | 0 | (0) | 0 | (0) | 0 | (0) | 2 | (6) |  |
| CRT after ESD | 0 | (0) | 3 | (4) | 13 | (32) | 16 | (49) |  |
| ESD alone | 65 | (100) | 71 | (96) | 27 | (68) | 4 | (12) |  |
| Venous invasion, positive (%) | 0 | (0) | 3 | (4) | 1 | (3) | 6 | (18) | <0.01 |
| Lymphatic invasion, positive (%) | 0 | (0) | 2 | (3) | 9 | (23) | 6 | (18) | <0.01 |
| LN metastasis, positive | 0 | (0) | 0 | (0) | 1 | (3) | 1 | (3) | 0.27 |

Ce, cervical esophagus; Ut, upper thoracic esophagus; Mt, mid-thoracic esophagus; Lt, lower thoracic esophagus; Ae, abdominal esophagus; ESD, endoscopic submucosal dissection; CRT, chemoradiotherapy; SM, submucosa; LN, lymph node

Supplementary table 2. Clinicopathological characteristics of patients with pT1b-SM cases

| **Characteristics** | **pT1b-SM1**  **n=30** | | **pT1b-SM2/3**  **n=67** | | ***P*-value** |
| --- | --- | --- | --- | --- | --- |
| Age, mean±SD, years | 69±8 | | 67±13 | | 0.73 |
| Sex, male (%) | 27 | (90) | 52 | (78) | 0.14 |
| Tumor size, mm, mean ± SD | 33±20 | | 33±18 | | 0.86 |
| Macroscopic type (%) |  |  |  |  | 0.48 |
| 0-I | 0 | (0) | 3 | (4) |  |
| 0-Ⅱa | 1 | (3) | 3 | (4) |  |
| 0-Ⅱb | 0 | (0) | 0 | (0) |  |
| 0-Ⅱc | 29 | (97) | 61 | (92) |  |
| Tumor location (%) |  |  |  |  | 0.67 |
| Ce/Ut | 8 | (27) | 13 | (20) |  |
| Mt | 14 | (46) | 37 | (55) |  |
| Lt/Ae | 8 | (27) | 17 | (25) |  |
| Circumferential range (%) |  |  |  |  | 0.76 |
| <2/3 | 23 | (77) | 50 | (75) |  |
| ≥2/3 | 4 | (13) | 7 | (10) |  |
| Whole circumference | 3 | (10) | 10 | (15) |  |
| Treatment (%) |  |  |  |  | 0.21 |
| Surgery alone | 8 | (27) | 30 | (45) |  |
| Surgery after ESD | 2 | (6) | 5 | (7) |  |
| CRT after ESD | 15 | (50) | 23 | (34) |  |
| ESD alone | 5 | (17) | 9 | (14) |  |
| Venous invasion, positive (%) | 2 | (7) | 22 | (33) | <0.01 |
| Lymphatic invasion, positive (%) | 10 | (33) | 25 | (37) | 0.71 |
| LN metastasis, positive | 2 | (7) | 20 | (30) | 0.01 |

Ce, cervical esophagus; Ut, upper thoracic esophagus; Mt, mid-thoracic esophagus; Lt, lower thoracic esophagus; Ae, abdominal esophagus; ESD, endoscopic submucosal dissection; CRT, chemoradiotherapy; SM, submucosa; LN, lymph node

Supplementary table 3. Clinicopathological characteristics of patients with pT1b-SM cases who underwent surgery

| **Characteristics** | **LN metastasis** | | | | | | ***P-*value** |
| --- | --- | --- | --- | --- | --- | --- | --- |
|  | **(+)**  **n=15** | | | **(-)**  **n=30** | | |  |
| Age, mean±SD, years | 64±9 | | | 62±9 | | | 0.63 |
| Sex, male (%) | 12 | (80) | | 19 | | (63) | 0.25 |
| Tumor size, mm, mean ± SD | 39±16 | | | 29±15 | | | 0.06 |
| Macroscopic type (%) |  | |  | |  | | 0.59 |
| 0-I | 1 | (7) | | 2 | | (7) |  |
| 0-Ⅱa | 0 | (0) | | 2 | | (7) |  |
| 0-Ⅱc | 14 | (93) | | 26 | | (86) |  |
| Tumor location (%) |  |  | |  | |  | 0.92 |
| Ce/Ut | 1 | (7) | | 3 | | (10) |  |
| Mt | 9 | (60) | | 18 | | (60) |  |
| Lt/Ae | 5 | (33) | | 9 | | (30) |  |
| Circumferential range (%) |  |  | |  | |  | 0.44 |
| <2/3 | 9 | (60) | | 22 | | (73) |  |
| ≥2/3 | 1 | (7) | | 3 | | (10) |  |
| Whole circumference | 5 | (33) | | 5 | | (17) |  |
| Color tone, reddish (%) | 8 | (53) | | 20 | | (67) | 0.38 |
| SMT like appearance (%) | 2 | (13) | | 9 | | (30) | 0.22 |
| Dominant histological type, poorly (%) | 4 | (27) | | 11 | | (37) | 0.27 |
| Depth of tumor invasion (%) |  |  | |  | |  | 0.08 |
| SM1 | 1 | (7) | | 9 | | (30) |  |
| SM2/SM3 | 14 | (93) | | 21 | | (70) |  |
| INF |  |  | |  | |  | 0.28 |
| a | 1 | (7) | | 7 | | (23) |  |
| b | 14 | (93) | | 22 | | (73) |  |
| c | 0 | (0) | | 1 | | (4) |  |
| Venous invasion, positive (%) | 4 | (27) | | 6 | | (20) | 0.61 |
| Lymphatic invasion, positive (%) | 10 | (67) | | 6 | | (20) | <0.01 |
| Treatment (%) |  |  | |  | |  | 0.24 |
| Surgery after ESD | 1 | (7) | | 6 | | (20) |  |
| Surgery alone | 14 | (93) | | 24 | | (80) |  |

Ce, cervical esophagus; Ut, upper thoracic esophagus; Mt, mid-thoracic esophagus; Lt, lower thoracic esophagus; Ae, abdominal esophagus SMT, submucosal tumor; ESD, endoscopic submucosal dissection; CRT, chemoradiotherapy; SM, submucosa; INF, infiltrative growth pattern

Supplementary table 4. Clinicopathological characteristics of patients with pT1b-SM cases who underwent ESD

| **Characteristics** | **LN metastasis** | | | | | | ***P-*value** |
| --- | --- | --- | --- | --- | --- | --- | --- |
|  | **(+)**  **n=7** | | | **(-)**  **n=45** | | |  |
| Age, mean±SD, years | 73±7 | | | 71±13 | | | 0.83 |
| Sex, male (%) | 7 | (100) | | 41 | | (91) | 0.41 |
| Tumor size, mm, mean ± SD | 36±5 | | | 33±21 | | | 0.42 |
| Macroscopic type (%) |  | |  | |  | | 0.12 |
| 0-I | 0 | (0) | | 0 | | (0) |  |
| 0-Ⅱa | 1 | (14) | | 1 | | (2) |  |
| 0-Ⅱb | 0 | (0) | | 0 | | (0) |  |
| 0-Ⅱc | 6 | (86) | | 44 | | (98) |  |
| Tumor location (%) |  |  | |  | |  | 0.20 |
| Ce/Ut | 4 | (57) | | 13 | | (29) |  |
| Mt | 3 | (43) | | 21 | | (47) |  |
| Lt/Ae | 0 | (0) | | 11 | | (24) |  |
| Circumferential range (%) |  |  | |  | |  | 0.34 |
| <2/3 | 6 | (86) | | 36 | | (80) |  |
| ≥2/3 | 0 | (0) | | 7 | | (16) |  |
| Whole circumference | 1 | (14) | | 2 | | (4) |  |
| Color tone, reddish (%) | 6 | (86) | | 33 | | (73) | 0.48 |
| SMT like appearance (%) | 1 | (14) | | 6 | | (13) | 0.95 |
| Dominant histological type, poorly (%) | 2 | (29) | | 4 | | (9) | 0.13 |
| Depth of tumor invasion (%) |  |  | |  | |  | 0.16 |
| SM1 | 1 | (14) | | 19 | | (42) |  |
| SM2/SM3 | 6 | (86) | | 26 | | (58) |  |
| INF |  |  | |  | |  | 0.19 |
| a | 1 | (14) | | 18 | | (40) |  |
| b | 6 | (86) | | 27 | | (60) |  |
| c | 0 | (0) | | 0 | | (0) |  |
| Venous invasion, positive (%) | 3 | (43) | | 11 | | (24) | 0.31 |
| Lymphatic invasion, positive (%) | 5 | (71) | | 14 | | (31) | 0.04 |
| Treatment (%) |  |  | |  | |  | 0.31 |
| CRT after ESD | 4 | (57) | | 34 | | (76) |  |
| ESD alone | 3 | (43) | | 11 | | (24) |  |

Ce, cervical esophagus; Ut, upper thoracic esophagus; Mt, mid-thoracic esophagus; Lt, lower thoracic esophagus; Ae, abdominal esophagus SMT, submucosal tumor; ESD, endoscopic submucosal dissection; CRT, chemoradiotherapy; SM, submucosa; INF, infiltrative growth pattern
